# Defining and predicting disease progression in myeloproliferative neoplasms: a proposed biomarker-driven approach

**DOI:** 10.1038/s41375-026-03103-0

**Published:** 2026-09-01

**Authors:** Raajit K. Rampal, Richard Winneker, Andrew Kuykendall, Laura C. Michaelis, Ghaith Abu-Zeinah, Steffen Koschmieder, Jan Philipp Bewersdorf, Zi Yun Ng, Gabriella Hobbs, Bridget Marcellino, Linda Resar, Joseph Scandura, Anand Patel, John Mascarenhas, Aaron Gerds, Douglas Tremblay, Shane Mayack, Andriy Derkach, Florian H. Heidel

**Affiliations:** 1 Leukemia Service, Department of Medicine, Memorial Sloan Kettering Cancer Center, New York, NY USA; 2 Center for Hematologic Malignancies, Memorial Sloan Kettering Cancer Center, New York, NY USA; 3 MPN Research Foundation, Chicago, IL USA; 4 Malignant Hematology Department, H Lee Moffitt Cancer Center, Tampa, FL USA; 5 Division of Hematology and Oncology, Medical College of Wisconsin, Milwaukee, WI USA; 6 Weill Cornell Medical College, Division of Hematology and Medical Oncology, Weill Cornell Medicine, New York, NY USA; 7 Department of Hematology, Oncology, Hemostaseology, and Stem Cell Transplantation, University of Aachen, Aachen, Germany; 8 Section of Medical Oncology and Hematology, Department of Internal Medicine, Yale School of Medicine, New Haven, CT USA; 9 Department of Haematology, Guy’s and St Thomas’ NHS Foundation Trust, London, UK; 10 Division of Hematology and Oncology, Department of Medicine, Massachusetts General Hospital and Harvard Medical School, Boston, MA USA; 11 Tisch Cancer Institute, Icahn School of Medicine at Mount Sinai, New York, NY USA; 12 Division of Hematological Malignancies and Bone Marrow Transplantation, Sidney Kimmel Comprehensive Cancer Center, Johns Hopkins University, Baltimore, MD USA; 13 Section of Hematology and Oncology, Department of Medicine, University of Chicago, Chicago, IL USA; 14 Cleveland Clinic Taussig Cancer Institute, Cleveland, OH USA; 15 Hematology, Hemostasis, Oncology and Cell Therapy, Hannover Medical School (MHH), Hannover, Germany

**Keywords:** Haematological diseases, Cancer genomics

## Introduction

The development of precision medicine approaches in oncology has revolutionized clinical practice. Although definitions of precision medicine may vary, it is reasonably defined as the use of biomarkers to select a treatment approach for a patient to optimize the chance of response [1–3]. Key examples that define current standard practice include the use of genomics to guide therapy, such as FLT3 inhibitors in Acute Myeloid Leukemia (AML) with FLT3-ITD, and the use of the Oncotype Dx assay to assess the relative risk and benefit of chemotherapy in invasive Breast Cancer [4, 5]. The number of such precision approaches in oncology continues to rapidly expand [1].

By contrast, the use of precision medicine in more indolent malignancies and pre-malignant conditions presents a different set of challenges. In many such diseases, such as monoclonal gammopathy of uncertain significance (MGUS) [6, 7] or clonal hematopoiesis of indeterminate potential (CHIP) [8–10], the likelihood of progression may be highly heterogenous: precision medicine approaches are often aimed to first understand which patients are at highest risk of progression to more aggressive disease, rather than to necessarily immediately identify a specific therapeutic intervention. A key example of where a precision medicine strategy is needed is the Myeloproliferative Neoplasms (MPNs). MPNs are clonal stem cell malignancies, consisting of Polycythemia Vera (PV), Essential Thormbocythemia (ET), and Myelofibrosis (MF) [11–13]. These diseases are characterized by blood count abnormalities, organomegaly, symptom burden for patients, and increased risks of thrombotic and hemorrhagic events. However, a proportion of patients will progress to a more advanced stage of disease, which can include transformation of PV or ET to MF, or progression of any of these entities to myelodysplastic/myeloproliferative overlap syndromes (MDS/MPNs), or accelerated/blast phase MPN (AP/BP-MPN)/secondary acute myeloid leukemia (sAML) [14–16]. Progression is often only curable by allogeneic stem cell transplant, but only a minority of patients are able to undergo transplant [17]. In diseases like ET and PV, where clinical courses can span decades, clinicians require tools to reliably predict disease at risk for progression. Current clinical trials for ET and PV focus primarily on managing peripheral blood counts and, to some extent, reducing thrombotic events. Yet, disease progression remains foremost on the minds of patients and physicians, and the lack of prognostic capability, coupled with limited intervention options directed at disease progression, causes unacceptable levels of anxiety for patients and caregivers and minimizes the urgency of disease modification. It is clear that understanding and predicting disease progression in myeloproliferative neoplasms (MPNs) represents one of the most pressing challenges in the care of patients with MPNs. It is also important to recognize that these transformations represent only one aspect of disease progression (as discussed below), highlighting the critical need for a broader definition of progression in MPNs.

To address this critical gap, future research must establish standardized, feasible, biomarker-driven models that account for disease heterogeneity and guide therapies. Existing risk stratification models, such as IPSET and MIPSS-ET/PV, incorporate driver mutations in *JAK2, CALR, MPL* and high-risk mutations in *SF3B1, SRSF2, TP53*, but lack standardized approaches for molecular monitoring. Emerging data suggest that factors like change in variant allele frequency (VAF), acquisition of genomic abnormalities, bone marrow fibrosis, and inflammatory cytokines (e.g., TNF-α, IL-8, CXCL12) could predict or possibly represent disease progression, though their clinical utility has not been validated.

## The MPN progression research network (PRN)

**Fig. 1:**
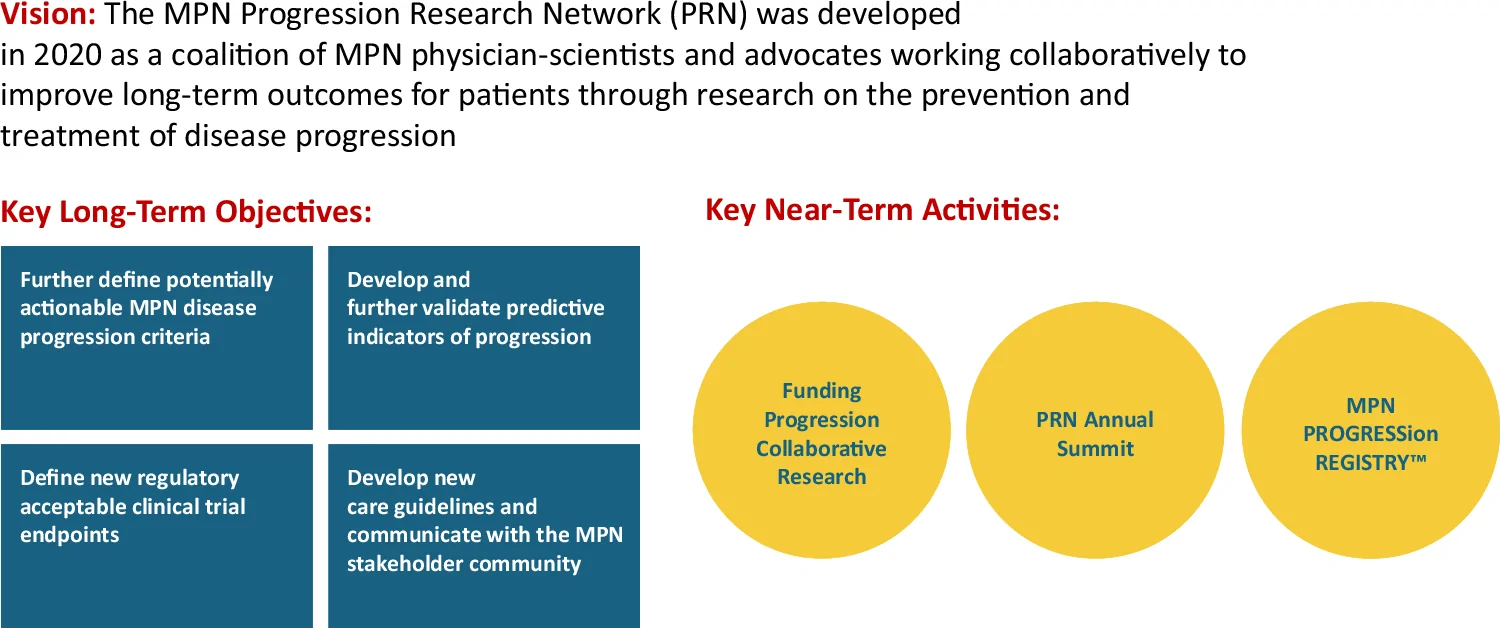
The MPN Progression Research Network (PRN). Long-term objectives and near-term activities.

The MPN Progression Research Network (PRN), established in September 2020 by the MPN Research Foundation, serves as a critical catalyst for facilitating collaboration on MPN research and improving patient outcomes through enhanced prevention and treatment of MPN disease progression. Annual PRN summits provide an important platform for discussing scientific advancements, identifying patient needs, and addressing research gaps (Fig. 1).

The objectives of these summits are actionable and time-sensitive: (1) refining MPN disease progression criteria through the study of clinical parameters and biomarkers, (2) validating predictive models for earlier detection and individualized intervention, (3) developing new clinical trial endpoints, and (4) establishing updated care guidelines. One major aim of the PRN is the development of a large, longitudinal, real-world evidence-based MPN patient registry. This manuscript summarizes the current understanding of progression and provides a concrete roadmap for future research on progression risk and early detection using clinical and biological parameters.

## Summary of clinical and biological factors in MPN disease progression

“Disease progression” in MPNs generally refers to uncontrolled disease marked by worsening symptoms, cytopenias, splenomegaly, thrombotic/hemorrhagic events, and/or transition to more aggressive myeloid malignancies [18] (Table 1). More formally, the International Working Group for Myelofibrosis Research and Treatment (IWG-MRT) defines progression in ET and PV as “transformation into post-PV myelofibrosis (MF), myelodysplastic syndrome (MDS) or acute myeloid leukemia (AML)” [19]. Criteria for MF transformation include prior confirmed ET or PV diagnosis (based on World Health Organization (WHO) standard), grade 2-3 bone marrow fibrosis (on the 0- 3 European Consensus scale), and at least two of the following: anemia, leukoerythroblastosis, increased LDH (for post-ET MF), splenomegaly (increasing or new), or constitutional symptoms. These definitions are mirrored in the 2022 International Consensus Classification (ICC) of myeloid neoplasms [20]. Progression to MDS/MPN or AP/BP-MPN is inferred based on WHO/ICC criteria for MDS/MPN and AML in patients with pre-existing ET or PV. ET-to-PV phenotypic transitions, which appear to be uncommon events, have been described [21, 22], potentially reflecting true biological change (loss of *JAK2*V617F heterozygosity giving rise to biallelic clones with erythroid bias) [23] or masked PV, and might also be considered progression events in the appropriate clinical and biological context.

**Table 1:**
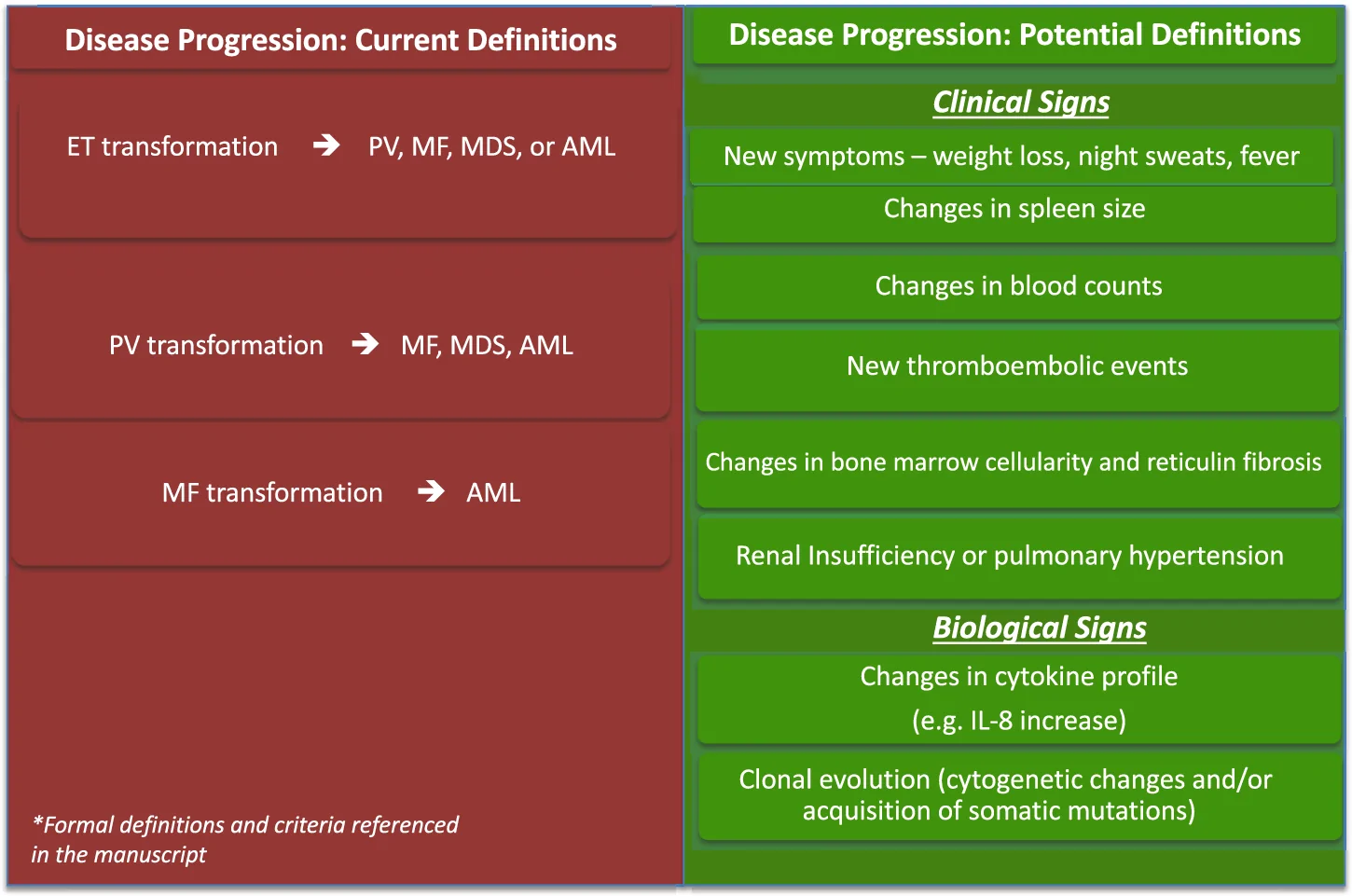
Progression events in Myeloproliferative Neoplasms.

Despite being driven by a single driver mutation (in *JAK2*, *CALR,* or *MPL)* in most cases, ET and PV exhibit marked phenotypic and clinical heterogeneity. Thus, the biology of MPNs likely involves an intricate interplay of genetic, epigenetic, microenvironmental, and immune/inflammatory signaling abnormalities [24]. However, the driver mutation itself carries prognostic significance, with multiple independent studies showing *that CALR-* and *MPL-*mutated ET is more likely to progress to MF and has a lower thrombotic risk than *JAK2*-mutated ET [25–27]. Existing ET risk models (IPSET-Thrombosis, IPSET-Survival, triple-A) primarily predict thrombosis or survival, not progression [28–30]. Similarly, the European LeukemiaNet (ELN) PV model stratifies patients into binary Low/High-risk for thrombosis according to age>60 and/or thrombosis history [31] and does not inform MF progression risk [32].

Clinical and molecular risk factors linked to MPN progression include: advanced age; prior thrombosis; leukocytosis (notably elevated absolute neutrophil count (ANC), lower absolute lymphocyte count (ALC), and higher neutrophil-to-lymphocyte ratio (NLR) [33]; driver mutation type and allele burden; co-mutations in epigenetic (e.g. *EZH2, IDH1/2*), RNA splicing (e.g. *SF3B1*, *U2AF1*, and *SRSF2*), and transcription factor genes (e.g. *TP53*, *RUNX1*, and *IKZF1*) [34–37]; cytogenetic abnormalities [38]; and, elevated proinflammatory markers [39] (Table 2).

**Table 2:** Putative and established risk factors for disease progression in MPNs.

Category	Specific Factor	Details/Evidence
**Demographics**	Advanced age	Associated with progression across MPN subtypes
	Male sex	Associated with progression in some studies
**Clinical Features**	Prior thrombosis	Risk factor for progression in REVEAL study
	Disease duration (longer)	Associated with progression in MOST and REVEAL studies
	Constitutional symptoms	Weight loss, night sweats, and fever indicate transformation
	Splenomegaly (new/increasing)	Part of formal MF transformation criteria
	Renal insufficiency	May signify disease-related organ damage
	Pulmonary hypertension	May signify disease-related organ damage
**Laboratory Parameters**	Leukocytosis	Higher WBC counts associated with progression
	Elevated absolute neutrophil count (ANC)	Component of predictive models
	Low absolute lymphocyte count (ALC < 1.7 × 10^9^/L)	Identified in triple-A risk model
	High neutrophil-to-lymphocyte ratio (NLR)	Associated with mortality and progression
	Anemia	Part of MF transformation criteria
	Leukoerythroblastosis	Part of MF transformation criteria
	Hematocrit <45% (in PV)	Risk factor identified in REVEAL study
**Driver Mutations**	*JAK2*V617F genotype	Higher thrombotic risk vs CALR/MPL; associated with ET-to-PV progression
	*CALR* mutation (vs JAK2)	More likely to progress to MF but lower thrombotic risk
	*MPL* mutation (vs JAK2)	More likely to progress to MF but lower thrombotic risk
	*CALR* Type 1 vs Type 2	Type 1 associated with higher MF risk
**Mutation Burden**	*JAK2*V617F VAF > 50%	Associated with progression and thrombotic risk
	*JAK2*V617F VAF increase	Longitudinal increase associated with progression
	Higher *CALR* mutant VAF	Associated with increased MF progression risk
	Loss of *JAK2*V617F heterozygosity	May drive ET-to-PV phenotypic transition
**Co-mutations**	*SF3B1*, *SRSF2*, *U2AF1*, *TP53*	Associated with adverse outcomes or included in MIPSS-ET/PV scores
	IDH1/2, ASXL1, EZH2, RUNX1, IKZF1	Associated with progression or adverse outcomes
**Cytogenetics**	+1q, del(17p), Complex karyotype	Implicate TP53 disruption or associated with adverse outcomes
**Bone Marrow Features**	Grade 2-3 fibrosis, cellularity, reticulin	Required for MF diagnosis or associated with progression
	Megakaryocyte morphology changes	May predict progression
**Inflammatory Markers**	Elevated TNF-α, IL-6, IL-1β, IL-2, IL-8, SDF-1, TGF-β	Correlate with progression or reduced survival
**Cellular Markers**	Elevated CD34+ cells, platelet transcriptomics, microRNAs	Potential biomarkers or implicated in progression
**Microenvironment**	VEGF, SDF-1, stem cell niche location	Reflect tumor niche or bone marrow microenvironment
**New Abnormalities**	New mutations, cytogenetic abnormalities, clonal evolution	Associated with progression

The multiplicity and interdependence of these risk factors and markers underlie heterogeneity of disease and complicate prognostication and therapeutic decision-making—challenges that demand coordinated solutions. Real-world data provide clinical insights that must be leveraged for clinical disease predictors. In the prospective Myelofibrosis and Essential Thrombocythemia Observational Study (MOST), patients with ET over 60 years of age or with a history of thromboembolic events, or who were receiving ET-directed therapy, demonstrated clear patterns that could inform clinical practice. Of the 1237 patients analyzed [40], 4.3% (53/1237) of ET patients progressed to MF. Patients with disease progression had longer disease duration, higher WBC counts, and lower hemoglobin. Using data from the Mayo Clinic MPN database, researchers have devised the triple-A risk model [30], which identifies low ALC ( < 1.7 ×10^9^/L), along with age and ANC, as predictive of poor survival. High ANC/ALC, also known as the NLR, has been associated with mortality [41]. The biology behind ALC as an adverse predictor in MPNs was recently elucidated [15], and it explains why the NLR may be a useful biomarker; however, its role in progression remains unclear. In the REVEAL study, 6.7% of PV patients progressed to MF over four years. Risk factors included longer disease duration, prior thrombosis, hematocrit <45%, leukocytosis, and higher *JAK2*V617F VAF [40].

For the treating physician, the development of new symptoms such as weight loss, night sweats, and fever may indicate transformation to MF or accelerated/blast phase disease. The presence of at least two of these symptoms meets the minor criteria for diagnosing post-PV or post-ET MF [19, 20] and may also be present in MPN-AP/BP. As in other hematologic malignancies, these symptoms likely reflect underlying cytokine dysregulation (e.g., TNF-α, IL-6, IL-1β), which may serve as potential biomarkers of disease activity. Additional clinical features—such as renal insufficiency or pulmonary hypertension—though not formal progression criteria, can signify disease-related organ damage and complicate management [42–45]. Along with routine monitoring of symptoms, blood counts with differential, and clinical events like new thromboembolism, physicians should also be aware of ancillary findings that may mark clinical worsening.

Bone marrow (BM) features also provide prognostic value. While BM cellularity does not predict survival in ET/PV, increased BM cellularity and high reticulin fibrosis are associated with progression to MF and AML [46]. In MF, an early study had demonstrated that patients with MF who had a hypo- or normocellular BM exhibited significantly inferior survival compared with those with a hypercellular BM. A later study showed that, even though mean BM hypercellularity was significantly higher in post-PV MF (95%) than in primary MF (PMF) (66%), there was no survival difference between the two groups of patients [47]. Together, these results suggest that, in ET and PV, high BM cellularity and fibrosis are associated with progression to MF (and thus also AML), and that later, after MF is established, hypocellularity is associated with inferior survival. However, one needs to acknowledge the heterogenous nature of fibrosis and inter-observer variability of fibrosis grading when considering the results stated above.

Progression has also been linked to genetic factors. The mere presence of a specific mutation (driver or additional) can be associated with inferior survival (e.g., *TP53*-disrupting or mutations in spliceosome genes) [48]. However, there may be vast differences in VAF both between each mutation in a single patient and between different patients [49, 50]. Also, current routine NGS analysis of the mutations usually cannot definitely elucidate the exact clonal complexity (e.g., whether two given mutations occur in the same vs. two independent clones). There are convincing recent data, however, showing that *JAK2V617F* VAF reduction in PV, particularly when it reaches the extent of complete or deep molecular response, was significantly associated with increased progression-free and event-free survival [51]. Likewise, a higher *CALR* mutant VAF in ET before onset of treatment was associated with an increased risk of progression to MF, when compared to a lower *CALR* mutant VAF [52]. These data suggest that, at least for the main MPN driver mutations, a high VAF was associated with increased risk of progression to MF.

A growing array of clinical, molecular, and inflammatory biomarkers is implicated in MPN progression. The complexity of these interrelated factors underscores the need for integrated risk models and the identification of truly independent, actionable predictors to inform prevention and treatment strategies.

## Defining current knowledge gaps: critical areas requiring action

Although MIPSS-ET and -PV scores incorporate somatic mutations that aid prognostication in ET and PV, several critical challenges regarding clinical implementation must be addressed [53, 54]. These gaps represent missed opportunities to improve patient care and require research prioritization. Key areas demanding urgent investigation include:

### Expansion of prognostic mutation panels

Current models (MIPSS-ET and MIPSS-PV) identify only a limited set of “adverse” risk mutations (*SF3B1* and *SRSF2* in ET and *SRSF2* in PV for overall survival, *U2AF1* and *SF3B1* in ET for myelofibrosis-free survival, and *TP53* for leukemic transformation in ET) [53]. However, these mutations only occur in a small minority of patients with ET and PV, which raises the question whether additional mutations with adverse outcomes such as *IDH1/2, ASXL1*, and *TP53* should also be formally included in scores predicting progression risk among patients with ET and PV [53–56]. Broader prognostic tools exist but lack validated frameworks for clinical integration [56].

### Standardization of VAF monitoring

While *JAK2*V617F mutant clones in patients, and especially *JAK2*V*617F* VAF > 50% is linked to progression and venous thrombotic risk, the clinical benefit of serial VAF measurements is uncertain due to technical variability, lack of standardized testing guidelines, and scarcity of long-term studies [57–60]. Open questions in this area include the interval of repeated testing (e.g., annually), technical difficulties, and lack of standardization of VAF assessment (including both *JAK2* and other driver mutations such as *CALR*), and what mutations should be tested for (e.g., only driver mutations or comprehensive panel testing) [61, 62].

Current *JAK2V617F* testing is often limited to granulocytes and does not reflect clonal contributions from megakaryocytes or erythroid precursors. Furthermore, studies have shown that contributions of the *JAK2 V617F*-mutant clone to peripheral blood lineages can be highly variable between individual MPN patients. This heterogeneity may underlie the biological continuum between *JAK2V617F* -positive ET and PV [63, 64]. Similarly, platelet transcriptomic profiles and thrombo-inflammatory signatures suggest complex, underexplored mechanisms of progression and symptomatology [65, 66]. Further, different *CALR* mutations are linked to divergent prognoses—type 1 with higher MF risk and type 2 with minimal progression risk—yet current clinical management does not differentiate between them [61, 62]. The field thus needs standardized testing guidelines and validated frameworks for clinical integration.

### Comprehensive cytokine profiling

Inflammatory cytokines in the marrow microenvironment likely contribute to both progression and thrombosis, yet cytokine profiling remains underutilized in clinical practice. Elevated TNF-α, IL-8, IL-2, and other cytokines correlate with progression, but timing, specificity, and reproducibility of these assays need further evaluation [65, 67]. Similarly, bone marrow hematopoietic cells, mesenchymal stromal cells, monocytes, granulocytes, and platelets interact in an intricate manner to produce numerous inflammatory cytokines to provide a self-perpetuating and permissive microenvironment for disease evolution in MPN [65].

In addition to contributing to disease progression, elevated inflammatory mediators and endothelial dysfunction contribute to thrombogenesis in MPN and are influenced by the underlying driver mutations that enhance the thrombotic environment. It is unknown whether the development of a thrombotic event in MPN itself is a marker of progression, or whether the prognostic implications of thrombotic events are driven by the morbidity and mortality of the events themselves.

Other parameters such as leukocytosis are consistently associated with progression and thrombosis [55, 68]. However, thresholds for cytoreductive therapy vary widely (e.g., 10–25 ×10^9^/L), and the efficacy of WBC reduction in preventing thrombosis remains unproven [53].

Cytogenetic abnormalities, such as +1q and del(17p), implicate *TP53* disruption in disease progression [69]. More sensitive and novel detection methods beyond traditional molecular cytogenetics (i.e. Karyotyping, FISH) of peripheral blood samples are needed for routine clinical application.

### Enhanced bone marrow assessment

The role of BM morphology at the time of diagnosis and as a marker of disease progression and response to therapy is not clearly defined. Recognizing the importance of fibrosis in outcomes, the World Health Organization (WHO) defined pre-fibrotic MF as a distinct entity with better clinical outcomes compared to patients with overt MF but worse than patients with ET [70, 71].

The WHO diagnostic criteria list a BM examination as a major criterion for PV. Findings in baseline bone marrow samples, such as the presence of fibrosis, have been demonstrated to be associated with the risk of progression to MF [72]. However, how best to standardize and utilize diagnostic bone marrow features for prognosis or to predict progression remains unclear. For ET, there are some features, such as increasing fibrosis and megakaryocyte morphology (such as large size with hyperlobated, deeply folded, or so-called staghorn nuclei), which may be features associated with progression but are not quantified [73]. The grade of fibrosis in post-PV and post-ET MF also appears to be associated with clinical parameters such as anemia and outcomes [74]. Thus, while bone marrow fibrosis is prognostically relevant, its diagnostic utility and quantification remain subjective and poorly standardized. Ongoing efforts to utilize Machine Learning-based image analysis may improve reproducibility and risk stratification [75]. These knowledge gaps represent actionable research priorities that, if addressed, could transform patient care within the next 5 years.

## Proposed biological and biomarker studies: a framework for action

Validated biomarkers are not just desirable—they are essential for enabling precision medicine approaches to predicting outcomes, managing disease progression, and guiding therapy in MPNs— but are limited due to the disease’s chronic nature and low transformation rates. There is an important unmet need to identify biomarkers that align with relevant clinical endpoints, including overall survival (OS), progression-free survival (PFS), thrombosis-free survival (TFS), and, in combination, event-free survival (EFS).

Biomarkers can serve distinct roles. Prognostic biomarkers assess risk. For instance, age in adults is always an adverse prognostic factor for death. Prognostic biomarkers are helpful as stratifying variables or inclusion criteria for clinical trials to help enroll patients with predictable risk and balance these risks across multiple arms, when relevant. Predictive biomarkers, on the other hand, forecast response to specific therapies. For example, in lung cancer, cancer morphology, specific mutations (e.g., *EGFR*, *KRAS*, *BRAF*, etc.) and staining patterns (e.g., PD-L1 expression) are predictive of response to specific therapies [76]. These biomarkers attempt to match patients with specific therapies to which they are most likely to respond favorably. No predictive biomarkers have been validated in MPNs in part due to the significant time required to assess outcome endpoints such as OS in conjunction with the endpoints needed to evaluate drugs utilized for MF (e.g., spleen size reduction and symptom benefit). Targeted therapies such as JAK2 inhibitors designed to target the specific *JAK2V617F* mutation or CALR antibodies that disrupt mutant *CALR* function will require unique biomarkers [77]. Since MPN-AP/BP has particularly poor outcomes, a specific biomarker that predicts patients at risk for leukemic transformation, along with a therapy that mitigates this risk, would dramatically change the field. Another significant unmet challenge in the field is to identify monitoring biomarkers that report in real time whether a treatment is working for an individual patient by tracking progress toward improved outcomes. Validated monitoring biomarkers could serve as surrogate endpoints in clinical trials, reducing the time and number of patients required to assess new therapies. Biomarkers that identify patients at highest risk for thrombosis could also provide more “personalized” goals for patients with regard to hematocrit targets. As it stands today, the field is hampered by the absence of validated predictive and monitoring biomarkers.

MPN driver mutations (JAK2, CALR, or MPL) originate in hematopoietic clones and may precede clinical diagnosis by decades, complicating risk prediction. As compared to MDS and some forms of clonal hematopoiesis where deleterious mutations dramatically increase the risk of progression within two years, the chronic and frequently indolent course of MPN has rendered the identification of risk factors for progression particularly challenging. Genomic studies utilizing mathematical modeling reveal that MPN mutations may be acquired much earlier than ever anticipated, including *in utero* [58, 78]. These clones frequently do not lead to MPN until much later in life, underscoring the difficulties with studying progression in MPN. Nonetheless, studies from MPN parallel findings in MDS and demonstrate that the acquisition of specific, deleterious secondary mutations influences disease progression.

Elimination of the MPN stem cell remains a therapeutic goal, but measuring its contribution to blood cell production remains difficult with current methods. Indeed, a decrease in *JAK2V617F* VAF has been used in clinical trials to assess the efficacy of specific therapies, but few studies have validated this approach, although the MAJIC-PV Trial and PROUD-PV/CONTINUATION-PV trials showed clinical and EFS benefit associated with a reduction in VAF [79]. Most studies rely on the *JAK2V617F* VAF in whole blood, which primarily reflects VAF in granulocytes variably admixed with lymphocytes that rarely harbor MPN driver mutations. Because peripheral blood has few stem and progenitor cells, changes in whole blood allele frequencies will not indicate the precise changes in stem and progenitor cells. Mutant VAF tends to persist in most studies to date, and the ideal target VAF remains unclear. The contribution of mutated stem cells to mature blood cell populations (MPN stem cell fitness) can change with treatment and seems to reflect the virulence of the malignant clone [80]. Based on epidemiologic data, preventing the acquisition of non-driver co-occurring mutations seems likely to be beneficial, possibly preventing MPN progression and other sequelae of MPNs. Longitudinal genomic studies and single-cell technologies offer promise in characterizing clonal evolution and interactions with the BM and vascular niches [81–84].

Epigenetic and microenvironmental factors, including chromatin architecture and stem cell metabolism, may further define risk. Since MPN driver mutations arise years or decades before MPN diagnosis, other factors, including transcriptional regulation, epigenetic state, microenvironment, and underlying hematologic traits, may provide clues to MPN risk and progression [85–87]. Newer genome-wide technologies that allow for more detailed dissection of chromatin architecture could illuminate changes that are required for MPN development. Intriguing studies of stem cell niches suggest that mutant hematopoietic stem cells (HSCs) within the more quiescent, endosteal niches associate with more indolent phenotypes, such as ET, whereas HSCs within the sinusoidal niches are more proliferative and activate gene networks linked to oxidative phosphorylation associate with PV, further implicating the stem cell microenvironment as a critical feature of MPN phenotype [85]. Emerging studies from patients and preclinical models suggest that the stem cell state, stem cell niche, stem cell metabolism, and the overall state of the patient also contribute to MPN onset and progression [88].

Recommendations for priorities:

1. The development and validation of predictive biomarkers that forecast response to specific therapies—a capability that does not currently exist in MPNs despite urgent clinical need.
2. Creation of biomarker-based surrogate endpoints for clinical trials, reducing the time and number of patients required to assess new therapies.

Future efforts must focus on tools that move beyond static risk prediction to provide dynamic insights that drive better patient care. Prospective studies will be crucial to validate currently used biomarkers and explore novel predictors of progression risk (Table 3). Integrating these variables (clinical, cellular, and molecular) in dynamic models could improve prognostication and inform treatment decisions. In order to truly ascertain how to best modify the risk of progression, it is imperative that there be prolonged follow-up of MPN clinical trials to determine the biomarker response that correlates with clinical outcome.

**Table 3:** Proposed biological and biomarker studies to address progression knowledge gaps.

Study Category	Proposed Research	Knowledge Gap	Clinical Application
**Molecular/Genomic**	Serial VAF monitoring (JAK2, CALR, MPL); expanded mutation panels (IDH1/2, ASXL1, TP53); clonal evolution studies; lineage-specific testing	Lack of standardized molecular monitoring; limited adverse mutation assessment	Risk stratification; treatment response monitoring; enhanced prognostic scoring
**Inflammatory/Cytokine**	Standardized cytokine profiling (TNF-α, IL-6, IL-1β, IL-8, TGF-β); bone marrow microenvironment analysis; stem cell niche studies	Underutilized cytokine assessment; unclear microenvironment role	Disease activity biomarkers; progression prediction; therapeutic targeting
**Epigenetic**	Chromatin architecture analysis; circulating microRNA studies (miR-150, miR-451); stem cell metabolism assessment	Limited understanding of epigenetic regulation; unknown metabolic factors	Non-invasive monitoring; metabolic therapeutic targets; disease mechanism insight
**Cellular/Morphological**	AI-based fibrosis grading; megakaryocyte morphology quantification; CD34+ cell monitoring	Subjective fibrosis assessment; lack of standardized morphological criteria	Improved reproducibility; diagnostic utility; disease burden assessment
**Thrombotic/Platelet**	Platelet transcriptomic profiling; thrombotic event relationship analysis	Underexplored thrombotic mechanisms; unclear event-progression relationship	Thrombotic risk assessment; progression prediction
**Cytogenetic**	Enhanced detection methods for +1q, del(17p); TP53 pathway analysis	Limited sensitivity of current cytogenetic methods	Early high-risk detection; treatment selection
**Clinical Validation**	Biomarker validation against clinical endpoints (OS, PFS, TFS, EFS); dynamic risk models; surrogate endpoint studies	Lack of validated biomarkers; inadequate trial endpoints	Clinical trial design; real-time risk assessment; accelerated drug approval
**Registry/Real-World**	MPN Progression Registry; multi-stakeholder collaboration	Need for standardized data collection; lack of coordinated approach	Regulatory evidence; biomarker validation; clinical decision support

**Key Implementation Priorities:** Standardization of assays, prospective study design, regulatory engagement, multi-modal biomarker integration, extended follow-up, and advanced technology utilization.

In addition, there must be a concerted effort to improve collaboration and dialogue between investigators, patient advocates, pharmaceutical, and regulatory collaborators in clinical trial design. Often, we see an imbalance of input when it comes to protocol development, especially enrollment criteria and endpoints. The financial driving force to obtain regulatory approval for emerging therapeutics strengthens the communication channels between regulators and industry but can sideline investigators and patients in that process. Moreover, to find the path of least resistance to approval, patients who may benefit can be excluded from clinical trials. For example, analysis of traditional acute leukemia enrollment criteria has identified a gap between the safety profile for an investigational drug and the organ-based criteria that need to be met for inclusion [89, 90]. Ultimately, excluding patients with organ dysfunction does not reflect expected toxicities, based on prescription drug labels, prior experience, or reported AEs on the trials [91]. Interrogation of these potential discordances in MPN studies would serve to broaden enrollment and clinical applicability of prospective findings. Another key point is not to just consider, but rather to prioritize the inclusion of patients with MPN-AP/BP in early-phase studies given that they are oftentimes excluded from both chronic-phase MPN studies and AML studies [92]. Lastly, while the body of evidence continues to grow for meaningful clinical endpoints, studies that are already in development must continue to be bold and incorporate endpoints that accurately capture both disease control and patient-reported outcomes. A key example of this is the SURPASS-ET study, which had a robust composite primary endpoint incorporating hematologic parameters with symptomatic improvement over a sustained period [93]. However, a key issue continues to be that even multi-faceted clinical endpoints like those used in SURPASS-ET have not been established as true surrogates for overall survival.

## Proposed clinical studies: transforming trial design

Current clinical endpoints in MPN trials (e.g., blood counts in ET or PV, spleen volume or symptom burden in MF) inadequately capture disease modification and must be revised. Therefore, it is of vital importance that we move beyond these inadequate clinical parameters to clinical trial endpoints that are better surrogates for disease progression and overall survival. However, there is a lack of consensus on what these new endpoints should be, as validated data supporting such a surrogate do not yet exist [94, 95]. One lead candidate is the assessment of molecular response in high-risk MPNs. In lower-risk MPN, such as PV and ET, molecular response, as measured by reduction of driver mutation VAF, has been observed with therapeutics such as ruxolitinib and interferon [51, 96–100]; furthermore, achievement of a molecular response has been associated with improved OS and EFS [51, 96]. Over time, the molecular profile of an individual’s MPN may become more complex as it progresses, and suppression of the driver mutation may not be sufficient to prevent progression of disease [101, 102]. With that in mind, molecular evaluation of driver mutations alone may not be adequate to capture disease modification. A post-hoc analysis of the Phase II REFINE study of navitoclax in MF suggests that a reduction in the VAF of high-risk mutations beyond canonical driver mutations may be associated with improved OS [103].

We propose prospective evaluation of molecular response in canonical driver mutations (*JAK2*, *MPL*, *CALR*) in addition to high-risk mutations and chromosomal alterations as defined by MIPSS-PV, MIPSS-ET, MIPSS70 + v2.0, and MYSEC-mPM/kmPM models based on the disease phenotype [104–106]. Depth of molecular response of the driver mutation should be characterized as per the ELN response criteria [107]. Additionally, while acknowledging the evolving definition of high-risk mutations, we propose evaluating high-risk mutations using the same response scale as driver mutations, with the emergence of new high-risk mutations classified as molecular progression [48, 108].

Large-scale studies are needed to validate biomarkers of disease burden and risk—such as reduction in peripheral blood CD34+ cells, BM fibrosis, megakaryocyte spacing, and changes in inflammatory cytokines—against outcome measures and clinical endpoints like spleen volume, symptom burden, thrombohemorrhagic events, anemia response, and ultimately PFS and OS. Ideally, individual or composite biomarkers could serve as early predictors of meaningful clinical outcomes. While smaller prospective and retrospective studies have suggested such correlations, robust, prospective trials with pre-specified biologic endpoints and multi-stakeholder collaboration (coordinated sponsor, patient, clinician, scientist, and regulator input and agreement) are essential for validation.

## Regulatory considerations: accelerating therapeutic development

Developing therapies that mitigate MPN progression requires the establishment of a rigorous framework of validated biomarkers and endpoints. MF, a high-risk MPN with rapid progression in specific patient subsets, offers a unique opportunity to refine these regulatory criteria. This section explores the current state of regulatory considerations, emphasizing the importance of high-risk MF populations enriched for genetic and molecular progression markers and lessons learned from analogous hematological malignancies.

In ET and PV, clinical endpoints are currently based on response criteria proposed by the European LeukemiaNet (ELN). These criteria include achieving hematologic control, resolution of disease-related signs, and symptom improvement [109]. Retrospective analyses have suggested an association between achieving ELN-defined responses and reduced progression risk. For instance, in a study of 398 PV patients treated with cytoreductive therapy, achieving an ELN-defined response was linked to a significantly reduced risk of progression (hazard ratio 0.39, 95% CI, 0.17–0.90) [110]. However, this association was confounded by the retrospective design and the subjective nature of progression definitions. Molecular endpoints, such as a ≥ 50% reduction in *JAK2V617F* allele burden, have also shown promise in trials like the MAJIC-PV study as previously discussed, but these have yet to undergo formal regulatory validation [96].

Current clinical trials in MF predominantly rely on clinical scoring systems, such as the Dynamic International Prognostic Scoring System (DIPSS), to stratify risk. Although effective for broad risk assessment, these scores do not adequately capture genetic and molecular high-risk features, which are critical for identifying populations with accelerated disease progression. Patients with adverse cytogenetic abnormalities, such as deletions in chromosome 17p or mutations in high-risk genes like *ASXL1*, *SRSF2*, *U2AF1*, and *TP53*, exhibit more rapid disease courses [111–113]. Incorporating these molecular and genetic markers into clinical trial designs allows for a more focused evaluation of progression-related endpoints, such as progression-free survival (PFS).

Candidate biomarkers for progression in MF include reduction in *JAK2V617F* or *CALR* allelic burden and cytogenetic remission. In addition to established molecular and cytogenetic markers, several emerging biomarkers described below have shown potential for predicting progression in MPNs, particularly in identifying patients with proliferative MPNs at heightened risk of transformation to MF or sAML. Inflammatory cytokines, such as IL-8, TNF-α, and transforming growth factor-beta (TGF-β), have been correlated with disease progression in MPNs. For example, elevated IL-8 levels are associated with reduced overall survival and a higher likelihood of transformation to advanced disease [39]. Circulating microRNAs (e.g., miR-150 and miR-451) have also been implicated in progression [114]. While inflammatory cytokine signals drive MPN progression in preclinical models and are associated with worsening disease in patients, they are associated with diverse disease states and, as such, lack specificity as prognostic or predictive biomarkers for MPN progression. However, it remains possible that increases in inflammatory signals within a given patient could indicate progressive disease and be suitable for disease monitoring. Prospective validation of the association between such cytokine levels and disease progression and/or transformation will be crucial. If validated, cytokine biomarker monitoring would require standardized clinical tests to ensure reproducibility across health care systems, similar to hemoglobin A1c levels as a predictive biomarker for diabetes risk. The regulatory challenge with such biomarkers lies in demonstrating their consistency and specificity across diverse patient populations and linking them robustly to clinically meaningful outcomes like PFS, as well as establishing standardized, reproducible assays across laboratories and demonstrating clinically meaningful outcomes through robust longitudinal studies.

There are inherent challenges to gaining regulatory acceptance for these biomarkers in their ability to predict PFS. To gain regulatory acceptance, trials must confirm that interventions modulating these biomarkers effectively alter disease trajectory, linking changes in biomarker levels to reduced progression risk or improved survival. In addition, there may be a heterogeneous impact of selected biomarkers based on an agent’s mechanism of action, further complicating uniform interpretation of a biomarker’s predictive ability.

Lessons from multiple myeloma demonstrate the potential for regulatory acceptance of surrogate endpoints and highlight the need for high-quality trials and standardized biomarkers. Minimal residual disease (MRD) negativity has recently been accepted by the FDA as a surrogate endpoint likely to predict clinical benefit and support accelerated drug approval [39]. This was based on a robust meta-analysis of randomized trials demonstrating a strong correlation between MRD negativity at 12 months and PFS [115]. However, applying these principles to MPNs remains challenging not only because MRD remains undefined in MPNs, but also due to the limited number of high-quality randomized trials that systematically assess progression. Furthermore, the lack of standardized surrogate markers—comparable to MRD in myeloma—represents a major gap in the MPN field.

For MPNs, the diversity of subtypes and lack of uniform endpoints pose challenges for biomarker validation. One may be tempted to study each subtype of MPN separately from others; however, a decrease in the number of evaluable studies and patients will subsequently decrease the statistical power of surrogacy. In the same vein, the simple proposition of aggregating all subtypes in the study increases the risk of diluting the surrogacy effect with subtypes where the surrogacy of a candidate biomarker is not valid. The second challenge for the statistical data analysis is that there are several candidate biomarkers to consider for surrogacy. For instance, if *JAK2V617F* VAF is considered a top candidate for surrogacy, one would have to define at what time point measurements are taken and what the clinically meaningful change is to maximize statistical power. Moreover, standardized assays would need to be developed (comparable to standardization for *BCR::ABL1* quantification in CML).

Regulatory pathways for these biomarkers would involve a two-phased approach, establish prognostic relevance in early-phase studies regardless of the treatment, followed by the second step of integration of prospective large-number of randomized clinical studies to confirm predictive value (effect on PFS or OS) in the same randomized trials. For instance, plotting changes in molecular markers, such as *JAK2V617F* VAF or BM fibrosis grades, against PFS could establish the predictive value of these endpoints.

Regulatory collaboration is essential to define the context of use for biomarkers and ensure clinical relevance. Agencies such as the FDA and EMA play a critical role in aligning biomarker development with regulatory requirements. Key steps include assay standardization, demonstration of clinical validity, and meta-analyses aggregating data across studies to establish these surrogate endpoints for progression-focused trials. Incorporating validated biomarkers into regulatory frameworks could accelerate therapeutic development aimed at mitigating progression risk in MPNs while ensuring scientific and clinical rigor.

Advancing biomarker and surrogate endpoint validation in MPNs requires a strong partnership among academic institutions, industry stakeholders, and regulatory bodies. Standardized criteria for molecular response thresholds, fibrosis grading, and cytogenetic evaluation are also fundamental. Enhanced data sharing from industry-sponsored trials would support cross-study analyses and strengthen the evidence base for clinical decision-making. Early regulatory engagement ensures alignment with regulatory standards and facilitates smoother approval pathways for the integration of novel endpoints.

Prospective clinical trials testing diverse therapies**—**including JAK inhibitors, epigenetic modulators, or immunotherapies**—**should incorporate candidate biomarkers and evaluate endpoints such as molecular responses, fibrosis dynamics, and PFS. Establishing these as validated surrogate endpoints requires consistent validation across studies and sustained stakeholder collaboration. Such coordinated efforts will bridge the gap between trial outcomes and regulatory approval, expediting the delivery of transformative therapies that enhance prognosis and quality of life for MPN patients. As a conceptual example, consider whether trials that enroll *JAK2*V617F-mutated ET and PV patients require serial VAF assessments performed at 6, 12, and 24 months. These changes would then be prospectively correlated with endpoints including thrombosis-free survival and PFS. Achievement of a ≥ 50% reduction in JAK2*V617F* VAF, for instance, could serve as a candidate surrogate endpoint, with its predictive value confirmed across multiple trials. Similarly, in MF, serial bone marrow assessments could evaluate fibrosis grade reduction as a candidate surrogate for PFS and OS, with at least one grade improvement at 24 months representing a clinically meaningful threshold for validation.

## Real-world data and registries: building the evidence base

Longitudinal real-world data (RWD) from patient registries offer an immediate pathway for establishing regulatory-acceptable real-world evidence (RWE) to support surrogate endpoint development. The MPN Research Foundation launched the MPN PROGRESSion registry^TM^ (NCT07362225) in September 2025. This national, patient-centric research initiative tracks the natural history of ET, PV, and MF, with a focus on disease progression. The Registry was developed specifically to transform MPN research from retrospective observational studies to predictive science, thereby addressing major knowledge gaps surrounding the natural history, biologic drivers, and clinical heterogeneity of MPN progression. This represents a critical opportunity to capture high-quality standardized molecular and clinical data to support validation and regulatory acceptance of endpoints such as PFS. Collaboration with regulatory agencies, including the FDA and EMA, will ensure that the collected RWD aligns with regulatory requirements, expediting the integration of RWE into clinical trial designs and supporting accelerated approval processes for novel therapies.

Advancing therapies aimed at mitigating progression risks in MPNs demands a paradigm shift in biomarker and endpoint development. High-risk patient populations, identified through genetic and molecular profiling, provide an opportunity to assess progression-related outcomes, including PFS, within feasible clinical timeframes. Drawing lessons from other malignancies, the MPN research community must prioritize standardization, design enriched trials, and ensure early regulatory engagement. These coordinated efforts will bridge the gap between trial outcomes and regulatory approval, expediting the delivery of transformative therapies that enhance prognosis and quality of life for MPN patients. Prospective trials with extended follow-up and coordinated stakeholder engagement will be key to validating biomarkers and enabling disease-modifying therapies that improve outcomes for patients with MPNs.

## Conclusion: a call for immediate, coordinated action

Progression prediction in MPNs remains an unmet need. Current frameworks lack standardized biomarkers, reliable predictive models, and validated surrogate endpoints—gaps that result in suboptimal patient care and missed therapeutic opportunities. Therefore, precision medicine approaches are required to improve the outcomes of patients. Our summary recommendations for the MPN community are to:

1. Integrate clinical, molecular, and inflammatory parameters—such as VAF dynamics, cytokine profiles, and BM fibrosis—into dynamic prognostic models where appropriate.
2. Launch prospective trials with extended follow-up and coordinated stakeholder engagement to validate biomarkers and enable identification of disease-modifying therapies.
3. Establish standardized protocols for biomarker assessment and clinical trial endpoints.
4. Accelerate regulatory engagement to ensure rapid translation of research findings into clinical practice.
5. Build the largest prospective MPN patient registry to further address fundamental questions regarding disease progression.

The tools, knowledge, and collaborative networks exist to transform MPN care. What is required now is a focused collaborative effort amongst all stakeholders in the community (patients, caregivers, clinicians/researchers, and biopharma). Advancing biomarker-driven approaches is a clinical imperative that will enable earlier intervention, personalized therapeutic strategies, and ultimately, improved clinical outcomes for patients with MPNs at risk of progression.
